# An Interprofessional Approach to Aural Rehabilitation for Adults with Hearing Loss and Cognitive Concerns

**DOI:** 10.3390/audiolres14010014

**Published:** 2024-02-04

**Authors:** Kate Helms Tillery, Aparna Rao

**Affiliations:** Speech and Hearing Science, College of Health Solutions, Arizona State University, Tempe, AZ 85281, USA; ahelms@asu.edu

**Keywords:** cognition, dementia, mild cognitive impairment, cognitive impairment, scope of practice, hearing loss, aural rehabilitation

## Abstract

Individuals with hearing loss are at risk for cognitive decline. The traditional approach to audiological care does not typically involve a team approach that addresses cognitive concerns. While cognitive screening is within the scope of practice in audiology, audiologists are not typically trained in interpreting screening results or providing rehabilitation that supports cognitive health. However, as growing evidence shows that hearing loss is tied to cognitive decline, a team approach is required to support whole-person care. Speech–language pathologists, who specialize in optimizing communication, are best situated to collaborate with audiologists to provide holistic aural rehabilitation. Audiologists and speech–language pathologists who partner to support a client’s communication skills and social relationships play an important role in the life of an individual with hearing loss. In this perspective, we describe relevant background information about hearing loss and cognition and present an interprofessional approach to aural rehabilitation for adults with hearing loss who have cognitive concerns. We also discuss implications for future research.

## 1. Introduction

About 15% of Americans over the age of 18 report some difficulty hearing. The prevalence of hearing loss increases with age; approximately 25% of individuals in the 65–74 age range and 50% of those 75 or older have disabling hearing loss [1].

Hearing loss in older Americans is tied to difficulty functioning in everyday situations, such as doing grocery shopping, managing finances, and using the phone. It is also associated with reduced quality of life in several domains, including a sense of vitality, social functioning, emotional engagement, and mental health [2]. Unfortunately, individuals with hearing loss do not always report their hearing and communication difficulties or seek treatment. Less than 30% of those 70 and older who would benefit from hearing aids have ever actually used them [1]. Older adults with untreated hearing loss are at increased risk for cognitive decline [3,4], which can further disrupt their ability to participate fully in daily life [5].

Because the effects of untreated hearing loss can include cognitive decline [6], clinicians must be alert to signs of cognitive concerns [7], which occur along a continuum that spans normal aging, mild cognitive impairment, and dementia. To provide context, these conditions are summarized briefly below.

### 1.1. Continuum of Cognitive Decline

*Normal aging*: Normal healthy aging is accompanied by declines in memory and perception, which, in turn, may affect more complex processes. Starting in early adulthood, processing speed, attention, working memory, visuospatial abilities, and some executive functions, such as mental flexibility and inductive reasoning, decline in a linear fashion across the decades. On the other hand, crystallized intelligence, including vocabulary and general knowledge, generally increases over time [8].

Normal cognitive changes due to aging do not impede an individual’s ability to function in daily life. Older adults without cognitive impairment (or other major health concerns) should be able to complete such tasks as managing their health and medications, overseeing their finances, and participating in family and social activities [9].

*Mild cognitive impairment*: Mild cognitive impairment (MCI) is a greater-than-expected cognitive decline based on an individual’s age and education level. Symptoms do not interfere significantly with daily functioning [10]. MCI may occur secondary to other neurologic, neurodegenerative, or psychiatric disorders. Some cases of MCI may be related to reversible conditions, such as depression or medication side effects. Individuals may remain stable or regain normal functioning over time. Some are at increased risk of developing Alzheimer’s and other dementias [11].

MCI is classified according to its predominant effects on cognition. Amnestic MCI primarily affects memory. Symptoms include losing things, forgetting events and appointments, and having trouble coming up with names. Non-amnestic MCI affects other cognitive abilities, such as language, executive function, spatial sense, and visual perception [12].

The prevalence of MCI is difficult to determine, partly due to variable diagnostic criteria. For individuals 65 or older, estimates range from 3% to 42% [13]. The risk increases with age; approximately 25% of those over 80 have MCI [11].

*Dementia*: Dementia is a loss of cognitive functioning that interferes with everyday life. Alzheimer’s disease is the most common form of dementia, accounting for up to 80% of diagnosed individuals. Other types include vascular dementia, Lewy body dementia, frontotemporal dementia, and mixed dementia [14]. Reversible dementia may occur due to metabolic/medical conditions [15] or nutritional deficiencies [16].

Symptoms include difficulty remembering current or past events, trouble recognizing friends and family, inability to complete complex tasks, lack of orientation to time and place, and loss of communication skills. Individuals may also show impulsive behaviors, poor judgment, and difficulty regulating emotions. While symptoms can vary depending on the type of dementia, some symptoms are common across all types, such as communication problems, disorganization, and impulsivity. Overall, dementia is characterized by a clear decline in the ability to perform activities of daily living. Functional impairments, along with cognitive and behavioral changes, are observed [11].

Almost 10% of adults aged 65 years and older have dementia. The risk of dementia increases with age. It is estimated that 2% of adults aged 65–69 years have dementia, and the percentage increases to 30% of adults over 90 years of age [17].

### 1.2. Relationship between Hearing Loss and Cognition

The link between hearing loss and cognition has received widespread attention in the last decade. Untreated hearing loss in adults is associated with a higher risk of developing dementia [3,4,18]. Individuals with mild hearing loss are twice as likely to develop dementia than those with normal hearing [4], and the risk increases with increasing severity of hearing loss [19]. This link between hearing loss and cognitive decline [4] was found using regression models on pure tone thresholds and dementia diagnosis data obtained on a population-based cohort. Therefore, it is important to note that the link is correlational and not causative [20].

While the nature of the relationship between hearing loss and dementia remains obscure, experimental studies provide some evidence of a link between hearing loss and altered brain structure/function. Impoverished input to the brain caused by hearing loss leads to cortical reorganization and structural changes. Increased responses were found to visual stimuli in the auditory cortical areas of individuals with mild to moderate high-frequency hearing loss, compared to those with normal hearing sensitivity [21]. Magnetic resonance brain scans of individuals with mild to moderately severe high-frequency hearing loss showed accelerated rates of shrinkage of the areas of the brain responsible for speech and sound processing, memory, and sensory integration, compared with those with normal hearing [22].

Other hypothesized mechanisms for cognitive decline in the presence of hearing loss center on the effects of cognitive load and social isolation. With untreated hearing loss, certain cognitive processes are often overburdened because the brain must work harder to fill in missing information. This occurs at the expense of other cognitive and memory systems [23], which could ultimately lead to cognitive decline. Social isolation and untreated hearing loss are independently associated with cognitive decline [24,25]. Disentangling the relationship may be challenging, as diminished sensory input and decreased intellectual stimulation may both ultimately lead to reduced cognitive abilities.

Hearing loss remains one of the most modifiable risk factors for dementia [26]. Treatment in the form of amplification devices has the potential to prevent or slow the rate of cognitive decline. Individuals with hearing loss who used properly fitted hearing aids had no difference in cognitive decline compared with controls [27]. Hearing aid use was associated with a lower prevalence of dementia in participants who had moderate to severe hearing loss compared with those who did not use hearing aids [19]. Results of a recent multicenter randomized controlled trial revealed that hearing intervention may reduce cognitive change in older adults at increased risk for cognitive decline but not in those at decreased risk [28]. This result highlights the critical need for further research on several individual and health factors that may contribute to cognitive decline.

Beyond hearing loss, speech-in-noise (SIN) impairment may be linked with dementia [29]. Results of a population-based study showed that participants with poor SIN abilities had an increased risk of developing dementia compared to those with normal SIN abilities. The measurement of central auditory processing skills also showed similar results. Those with poor sentence recognition in the presence of ipsilateral competing messages and poor dichotic listening skills had a higher risk for clinical dementia [30,31].

### 1.3. Hearing Loss and Comorbidities in the Presence of Cognitive Decline

Other health conditions that frequently coexist with hearing loss include visual impairment and manual dexterity problems. Both of these conditions are also associated with MCI/dementia [32,33,34].

Vision loss, another modifiable risk factor for dementia [33], may affect cognition in the same way as hearing loss, compromising sensory input to the brain. The contribution of vision loss to lifetime risk for dementia may be smaller than that of hearing loss due to readily available effective treatments, such as prescription eyeglasses and cataract surgeries [32].

Manual dexterity, which involves the control and coordination of finger movements, requires sensorimotor integration and cognitive skills, such as attention and working memory. Problems with manual dexterity may be associated with cognitive decline [35]. Individuals with cognitive decline may show difficulty with fine motor tasks that require attention and short-term memory [36].

Both vision and dexterity issues impact aural rehabilitation for an individual with hearing loss. Specific considerations are discussed in the Section 2 below.

### 1.4. Scope of Practice Related to Cognition

All audiologists who work with older adults should be knowledgeable of the connections among hearing loss, cognitive impairment, speech perception in noise, central auditory processing, communication abilities, and overall well-being. Clinicians should be alert to signs of cognitive impairment or cognitive concerns expressed by the client, family, or caregivers, and conduct screenings as needed. A client or caregiver expressing concerns regarding thinking skills, forgetfulness, personality changes, or inappropriate behaviors may indicate a need for screening and diagnostic follow up.

Screening for cognitive impairment is within the scope of practice of audiologists. The American Speech Language Hearing Association’s Ad Hoc Committee on the Scope of Practice in Audiology (2018) [37] states:

“Clinical service delivery areas include all aspects of hearing, balance, and other related disorders that impact hearing and balance, including areas of tinnitus, cognition, and auditory processing for individuals across the lifespan. ….Additional screening measures of mental health and cognitive impairment should be used to assess, treat, and refer”.

While the audiological scope of practice includes cognitive screening, studies show that most audiologists do not integrate such screenings into their typical care plan [38]. In addition, the audiological scope of practice does not include intervention for cognitive concerns, and most audiologists do not have the training to work with clients or caregivers when cognitive concerns arise after screening [6,35].

One approach that we believe can address this problem adequately is a collaborative model involving speech–language pathology and audiology. Speech–language pathologists are well prepared to conduct cognitive screenings, assessments, and interventions for individuals with hearing loss [39].

ASHA’s document on Scope of Practice in Speech-language Pathology (2016) lists both cognition and hearing as areas of competency [39]:

“Service delivery areas include all aspects of communication and swallowing and related areas that impact communication and swallowing: speech production, fluency, language, cognition, voice, resonance, feeding, swallowing, and hearing”.

## 2. New Perspective: An Interprofessional Approach to Aural Rehabilitation

Aural rehabilitation is key to improving communication, promoting participation in social activities, and facilitating emotional well-being, especially for clients with both hearing loss and cognitive concerns. Audiologists and speech–language pathologists at the Arizona State University Speech and Hearing Clinic collaborate to provide team-based, person-centered aural rehabilitation that supports functional outcomes for clients and their families and caregivers. Students from both the audiology and the speech–language pathology programs are involved in interprofessional training and service provision.

Components of aural rehabilitation have traditionally included the selection and fitting of appropriate hearing technology, instruction in care and maintenance of devices, management of the listening environment and use of communication strategies and self-advocacy, personal adjustment counseling, and sometimes direct training of speech perception skills [40]. Aural rehabilitation may also include education and training for family members and caregivers [41].

In this section, we describe our perspectives on an interprofessional approach to aural rehabilitation for individuals with hearing loss and cognitive concerns. Our experiences in conducting cognitive screening, making referrals, selecting technology, fitting devices, and optimizing communication are discussed. When available, the relevant literature is summarized to provide an evidence base for our approach.

### 2.1. Cognitive Screening

In our clinic, we follow the guidelines outlined in the 2020 report from the United States Preventive Services Task Force [13]. This report indicates that there is insufficient evidence to support cognitive screening for individuals 65 or older who do not have cognitive concerns. Screening is helpful, however, for individuals who show signs of cognitive decline or who express concerns about their cognitive skills.

Candidates for cognitive screening include older adults who present with communication difficulties, attention or memory concerns, vestibular or balance problems, excessive difficulties perceiving speech in noise, or a family history of MCI/dementia. Adults with complex and chronic medical issues who may present with polypharmacy (defined as regular use of at least five medications, common in older adults) are also candidates for screening [42].

In the context of aural rehabilitation, the main goal of cognitive screening is to refer to a specialist when results indicate the need. The audiologist and speech–language pathologist work together to explain screening results to clients and their caregivers and, when indicated, encourage clients to follow up with a specialist. Screening results are taken into consideration when we design individualized aural rehabilitation plans.

Cognitive screening instruments can be used by both audiologists and speech–language pathologists. There are many cognitive screening tools available. The tools we have used in our clinic include:

- Mini Mental State Exam (MMSE) [43];
- Montreal Cognitive Assessment (MoCA) [44];
- Saint Louis University Mental Status Exam (SLUMS) [45];
- Cognivue Thrive [46].

These screeners serve only to identify individuals who may be at risk for cognitive concerns. Formal evaluations and diagnoses are carried out by the client’s care team, which may include a speech–language pathologist, primary care physician, neurologist, neuropsychologist, psychiatrist, caregivers, family members, and others.

It is critical to note that hearing loss can impact performance on instruments that rely on verbal communication to screen or test cognitive skills [47]. To reduce the likelihood of over-referral, we take hearing status into account before and during the test [48]. The screening is conducted in a quiet, distraction-free environment. Communication is optimized by ensuring that the client has access to as clear a signal as possible. This may involve the use of a personal amplifier, hearing aids, glasses, and/or visual (speechreading or text) cues.

In our clinic, we have experienced a range of individual client perspectives regarding cognitive decline. Some clients report neurological diagnoses related to MCI or dementia at the time of the case history interview. Some openly express concern about their thinking or memory skills and state an interest in completing a cognitive screener. Others agree to complete a screener once we discuss the topic of cognitive health during the aural rehabilitation process. A few clients are very sensitive to the stigma associated with cognitive decline and are straightforward about their desire not to discuss cognitive issues, even though they present with risk factors (e.g., hypertension, head injury, social isolation, family history) or show symptoms of cognitive decline. Family members or caregivers who are willing to openly discuss their loved one’s cognitive concerns are instrumental in assessment planning and decision making regarding potential hearing technology and aural rehabilitation steps.

We have found that the screening can be introduced in a gentle, low-key manner in the context of aural rehabilitation. After the hearing loss is diagnosed, we explain to the client that cognitive screening can help us understand their attention, memory, and cognitive processing needs, which will guide our selection of hearing aids and inform our choice of topics addressed in communication training. Clients are reminded that this is a screening tool only and that decisions regarding further steps should be made in consultation with their primary care provider, specialists, caregivers, and family members. Throughout this conversation and screening procedures, we ensure that the client has access to a personal amplifier, any needed visual aids, such as glasses, and good lighting. We use Clear Speech strategies [49] and/or speech-to-text transcription to support clear communication.

### 2.2. Hearing Technology

For those with permanent hearing loss, amplification through hearing aids is the standard treatment approach. When there are cognitive concerns, signal processing that includes slower release time with wide dynamic range compression may facilitate speech perception [50,51]. Other features that facilitate speech perception may include automatic programs with directivity and automatic T-coil activation.

For clients in our clinic with diagnosed or suspected cognitive issues, we usually consider the range of device options, from traditional hearing aids, to Hearing Assistive Technology (HAT) devices (such as a remote microphone), to the use of a personal amplifier. We are usually able to openly discuss visual acuity and dexterity skills in making decisions about amplification. For individuals with vision or dexterity concerns, custom ear molds may be preferred over non-custom domes that need to be replaced on a regular basis. Similarly, rechargeable instruments may be selected over hearing aids that require batteries. The use of a smartphone app to control volume may be more feasible than trying to manipulate a button on the hearing aid.

When programming hearing aids, we provide one automatic program for listening in quiet and in noise with automatic activation of features, such as directionality and noise reduction. If a client wants to have some control in a specific situation (for example, at a church that is looped), we provide a second T-coil program that they can switch to.

Written instructions on the use and care of hearing aids are provided in client-friendly language using a visually accessible font size. We use pictures and graphics to support understanding. Ample practice in the insertion and removal of hearing aids and changing of batteries and cerumen guards is provided. For some clients, it is necessary to revisit topics multiple times before they demonstrate an understanding of the care and use of their devices. In some cases, hearing aid retention systems are needed to prevent accidental removal and loss of hearing aids. A family member or caregiver is also trained in the maintenance and proper storage of devices, which has been shown to support device care and use [52]. We have found that it is very important to discuss the loss and damage warranty with the client and caregiver and extend the warranty period, whenever possible.

Clients with mobility concerns who are unable to participate in social events in person often remain connected to friends and family via their phones. In these instances, we often choose hearing aids that offer direct connectivity to their mobile phone via Bluetooth. Others have expressed the need to connect to an iPad or phone to listen to podcasts and other programming for entertainment. These options typically require extra training and support, as we like to encourage all listening and communication activities. When clients are comfortable using a phone and an app for hearing aid control, we have helped them with the app as well. Sometimes, the caregiver downloads the app on their phone to connect with the client’s hearing aids.

Whenever possible, we promote social engagement. Communication training is provided for specific situations. This training may include the use of HAT devices, such as a remote microphone in a lecture or a dining hall.

In cases of more severe cognitive decline, decisions regarding hearing amplification are made with greater input from the caregiver. Obtaining impressions for custom devices or earmolds for behind-the-ear hearing aids may be challenging. In one case, we decided to use a personal amplification device, rather than a traditional hearing aid. This individual lived in a memory care facility and did not have the support system needed for the use of traditional hearing aids. The client’s ability to use a personal amplifier was demonstrated and confirmed in the clinic. The volume control for comfortable listening was noted for future use. The client’s caregiver was counseled on the use and maintenance of the device.

### 2.3. Instruction in Environmental Management, Communication Strategies, and Self Advocacy

In our clinic, instruction in environmental management, communication strategies, and self-advocacy is provided by a speech–language pathologist. This session usually occurs immediately after the audiological appointment to reduce the transportation and travel demands on clients and their caregivers. In the case of cognitive decline, information is shared with caregivers, as well as with clients. Multiple appointments are sometimes necessary to accommodate for attention and fatigue concerns. Teletherapy sessions are offered as needed. For all topics covered, printed information in client-friendly language with visually accessible font is provided for clients and caregivers to take home. Teaching strategies such as explicit categorization (labeling each topic before discussing it), repeating important information, and breaking complex information into smaller chunks [53] are effective ways to support clients’ ability to understand and remember information. Inviting clients to ask questions and encouraging them to restate what they have learned are helpful ways to confirm their understanding of instructions [54].

The appointment starts with an interview focused on identifying the client’s daily communication needs and experiences. Ethnographic interviewing techniques [55], such as asking open-ended questions, restating the client’s message, and providing opportunities for the client to confirm or correct our summary, are helpful for building rapport and gaining insights into their communication and cognitive status. Our aim is to learn about the client’s home environment, the people they talk with regularly, the kinds of sounds they need to be aware of, and the types of communication situations and challenges they encounter during the week (e.g., medical appointments, family gatherings, phone calls, etc.). We also learn what type of telephone they use. This information guides goal planning and the selection of topics addressed in our sessions.

To address management of the listening environment, we individualize information based on the client’s living situation. For clients who live with family, we educate family members on the importance of reducing background noise during meal times and other gatherings. Family members are advised to hold conversations away from noise in a well-lit area to allow for speechreading cues. In Arizona, where tile floors are common, families are encouraged to use area rugs and other sound-absorbing materials to reduce the effects of reverberation. For clients who live alone, we discuss the importance of avoiding or reducing background noise when talking on the phone or with neighbors. Speech-to-text apps, such as Live Transcribe (for Android) and NALScribe (for iPhones), are introduced and demonstrated. Those who report difficulty hearing safety sounds are put in touch with the Arizona Commission for the Deaf and the Hard of Hearing, which provides accessible equipment, such as lighted doorbells and captioned telephones.

To support communication, clients and their caregivers are given information about strategies to avoid and repair communication breakdowns. Instructions are provided regarding how to get the listener’s attention before talking: face the listener, make sure speechreading cues are available, speak in short sentences, and confirm the listener’s comprehension of the message. Caregivers are also taught Clear Speech techniques [49], such as enunciating words, pausing occasionally to provide processing time, and using natural intonation and gestures. Clients are instructed in the benefits of posing specific questions, rather than asking “What?” when they miss information. These strategies are described and modeled, and clients and caregivers are asked to practice using them in the clinic to ensure that they understand the procedures before going home. When clients are able to report on previous communication breakdowns, we troubleshoot the incident, discuss possible solutions, and sometimes even role-play the scenario using appropriate communication strategies. Research has shown this approach to be effective in managing the effects of more severe cognitive decline [56].

To support telephone communication, clients are given the opportunity to practice holding a conversation over the phone while in the clinic. This allows the client to gain experience connecting their hearing aids to their phone, adjusting the volume, and using communication and self-advocacy strategies in a more challenging context.

To promote self-advocacy, clients are encouraged to disclose their hearing loss to conversational partners and give specific feedback about what they need the talker to do to ensure understanding. The clinician works with the client to develop a self-advocacy script that they would like to use in community settings, such as at the grocery store or medical appointments. The script is typically short, such as “I’m hard of hearing. Please face me and speak clearly so I can understand you”. The client is asked to practice using the script while the clinician simulates ineffective communication behaviors, such as turning away or looking down while talking. Many of our clients report that practicing with the script helps them use it more readily in actual communication breakdowns.

### 2.4. Counseling: Supporting Emotional Well-Being of Clients and Caregivers

The combined effects of hearing loss and cognitive impairment can lead to anxiety and stress for both clients and their caregivers. In her work describing effective supports for caregivers of adults with communication disorders, Joan Payne [57] recommends that clinicians take time to talk with clients and caregivers so that both parties have an opportunity to express their needs and concerns. Providing a safe environment in which to express thoughts and emotions, beyond those related specifically to device programming, is beneficial for clients and their family members and caregivers.

In our clinic, we have the ability to schedule back-to-back audiology and speech–language pathology sessions for our clients with cognitive decline, which provides ample opportunities for discussion of thoughts, feelings, and concerns. At the beginning of both the audiology and the speech–language pathology sessions, clients and caregivers are invited to express their needs and ask questions, which helps set the goals for the appointment.

For clients who are from culturally and/or linguistically diverse communities, we have access to a telephone interpreting service to ensure clear communication. Ethnographic interviewing techniques are particularly helpful in identifying concerns of caregivers from diverse backgrounds. When possible, we provide printed materials in the client’s preferred language. We use a range of cultural responsiveness resources [58,59,60,61] to remain mindful of differing views on health, disability, and family structures and to acknowledge the unique perspectives and values of each individual.

When clients or caregivers express emotional content, counseling strategies such as reflective silence, naming emotions, and affirmations [62] are effective ways to hold space and provide a safe environment for processing strong feelings. On the rare occasion that clients express ambivalence or reluctance about participating in aural rehabilitation, motivational interviewing techniques can be useful [63]. Double-sided reflections, such as “On the one hand, you haven’t been wearing your hearing aids, and on the other hand, you want to hear your spouse better”, and hypothetical questions, such as “What do you think might happen if you tried your hearing aid during dinner tomorrow?”, can help clients identify and move past obstacles that are impeding their progress. When needed, we make referrals to professional counselors, support groups, consumer advocacy organizations, and community resources.

### 2.5. Lessons Learned

For adults with hearing loss who have cognitive concerns, aural rehabilitation involves addressing not only their hearing and cognitive needs but also their other wellness concerns [64]. Coordinated care provided by an interprofessional team is important. This approach requires planning and clear communication among all team members.

In our clinic, we have learned that the transition time between the audiology and speech–language pathology appointments is an important opportunity for the two providers to touch base and share information about the client’s needs. We have developed a one-page form summarizing the client’s technology (for example, Bluetooth, t-coil, availability of an app) that the audiologist hands off to the speech–language pathologist immediately before the aural rehabilitation appointment. We have also learned that weekly staffing and shared documentation in our electronic medical record are critical for ensuring continuity of care.

To address the overall health and wellness needs of our clients, we rely on a wide range of community resources. Family members, social workers, and audiology or speech–language pathology assistants may be helpful in supporting communication among the care team and coordinating services. Counselors and community health workers can provide emotional and physical support. Cognitive therapy, often available through private practitioners and university speech and hearing clinics, can support the maintenance of cognitive skills. Access to alerting devices and other assistive technologies may be available through community agencies.

To document outcomes, we have used the Client Oriented Scale of Improvement [65] to set client-specific communication goals and track perceived changes over time. The TELEGRAM tool has been useful in encouraging clients to consider a variety of communication need areas before setting goals [66]. Formulating goals that are specific and relevant to clients’ daily lives requires some guidance from the clinician and is important for measuring progress. For example, the specific goal “understanding my spouse during meals” is preferred because it is easier to track than a more general goal, such as “hearing my family”.

In general, we have found that successful outcomes can be supported by:

- Breaking complex information into smaller chunks;
- Sharing client-friendly printed information to support memory;
- Encouraging the practice of skills before the client leaves the clinic;
- Scheduling short breaks or multiple appointments to accommodate for attention and fatigue concerns and troubleshooting;
- Inviting caregivers to participate in appointments.

## 3. Conclusions

In order to effectively serve individuals with hearing loss and cognitive decline, we have proposed and described an interprofessional approach involving audiologists and speech–language pathologists. The knowledge and skills of both professionals are required to address communication and cognitive skills. The many relative strengths of this approach are described below, along with barriers we have encountered in the delivery of interprofessional services. While we expect that this approach will lead to improved outcomes, further research is needed to document them.

### 3.1. Relative Strengths of an Interprofessional Approach

Interprofessional collaboration between audiologists and speech–language pathologists affords a range of benefits for clients, professionals, and trainees. In a survey of practicing audiologists and speech–language pathologists, respondents from both disciplines placed a high value on interprofessional collaboration [67]. Reported advantages of teamwork included improved efficiency of clinical care, clearer communication, and an increased appreciation for the roles and responsibilities of the other profession [68]. In our clinic, audiologists and speech–language pathologists report that interprofessional collaboration has contributed to improved communication regarding client care. Clients have expressed appreciation for the integrated approach to clinical services.

Studies of interprofessional education indicate that audiology and speech–language pathology trainees who participate in team-based clinical experiences report benefits to both their knowledge base and skill set. In one study, students reported feeling more confident in their own clinical skills, more knowledgeable about their counterparts’ discipline, and more at ease when working in interprofessional settings [69]. Another study reported that students developed communication and consensus-building skills [70].

Anecdotally, speech–language pathology trainees in our program who participate in interprofessional experiences report an increased understanding of hearing loss and hearing technology, and audiology trainees report an increased appreciation for the value of communication training. Students in both disciplines report feeling more confident when communicating with their counterparts.

### 3.2. Barriers to an Interprofessional Approach

Individualized intervention with an interdisciplinary approach depends on the commitment of individuals involved, a significant investment of time, and support received from the institution. The main barrier in our clinic has been a lack of time to collaboratively plan for and participate in the tailored sessions for clients due to our varying schedules [71]. The cost of delivery of services is also a consideration, as we are expected to meet fiscal benchmarks in our clinic. When two professionals are working with one client collaboratively, the cost is higher. Services have to be covered via private pay, third-party payers, or grant funds received to treat individuals with limited income.

### 3.3. Implications for Future Research and Practice

We provide face-to-face care in a traditional university-based clinical setting and supplement it with teletherapy for individuals who experience health concerns or transportation issues that make it difficult for them to travel to our clinic. Positive effects of teletherapy have been reported both for clients with cognitive decline [72,73,74] and those with hearing loss [75,76,77], but there are no published studies examining outcomes of teletherapy for those with both hearing loss and cognitive decline. While our clients have expressed an appreciation for the option to receive services remotely, further research is needed to document the effects of teletherapy for individuals who have both concerns.

Another supplement to the traditional model of individual face-to-face services is group-based therapy. Group therapy models have been used successfully in speech–language pathology and audiology to provide individuals and caregivers with functional communication training and meaningful psychosocial support [78,79]. In the future, we would like to integrate group therapy into our services, but more research is needed to inform our development of effective group-based interventions for individuals who have both hearing loss and cognitive decline.

While perspectives such as the present article are a helpful way to share clinical experiences and insights, controlled research studies are necessary to guide the development of evidence-based approaches for this population and ensure the long-term sustainability of functional interventions. In the meantime, it is hoped that readers will find this information useful when designing their own functional approach to aural rehabilitation for older adults with cognitive concerns.

## Data Availability

No new data were created or analyzed in this study. Data sharing is not applicable to this article.
